# Barriers and Concerns that Contribute to Vaccine Hesitancy in Black, Indigenous, and People of Colour (BIPOC) Individuals in Ontario, Canada

**DOI:** 10.7759/cureus.63033

**Published:** 2024-06-24

**Authors:** Pria Nippak, Housne Begum, Wajiha Ahmed, Devi Santhikumar

**Affiliations:** 1 Health Services Management, Ted Rogers School of Management, Toronto Metropolitan University, Toronto, CAN

**Keywords:** coronavirus disease 2019, concerns about vaccinations, barriers to vaccinations, vaccine hesitancy, minority population, bipoc, covid-19

## Abstract

Background: Despite research demonstrating the effectiveness of COVID-19 vaccines, hesitancy is extremely common in minority communities. The purpose of this study was to identify key barriers and concerns that contribute to vaccine hesitancy in Black, Indigenous, and People of Colour (BIPOC) individuals and provide recommendations to address these barriers and concerns.

Methods: The study was an online cross-sectional survey conducted among 1491 BIPOC and Caucasian adults, recruited using social media networks in August-September 2021. The questionnaire consisted of five sections that probed concerns and attitudes contributing to COVID-19 vaccine hesitancy.

Results: Respondents were mostly Caucasian males (75.7%) and the average age was 29.1 years. A higher proportion of BIPOC respondents received both doses (50.6%) than Caucasian respondents (36.4%). Out of the unvaccinated, a higher percentage of BIPOC respondents did not plan on getting vaccinated (17.1%) compared to Caucasian respondents (4.2%). BIPOC respondents preferred the Pfizer-BioNTech (34.1%) vaccine whereas Caucasian respondents preferred AstraZeneca (29.3%). The biggest concern BIPOC and Caucasian respondents had with COVID-19 vaccines were side effects (56.6% vs 54.4%, respectively). BIPOC respondents identified dependability as the next biggest concern after side effects. A higher percentage of BIPOC respondents were against getting vaccinated against COVID-19 (16% vs 1.2%) compared to Caucasian respondents.

Conclusion: Among unvaccinated respondents, COVID-19 vaccine hesitancy was most evident in the BIPOC respondents compared to Caucasian respondents. Side effects, trustworthiness, and lack of information were identified as the three most common concerns surrounding vaccines in general. Increased accessibility to reliable and accurate vaccine information in various languages/dialects can raise awareness about COVID-19 vaccinations in BIPOC communities

## Introduction

Globally, the coronavirus disease 2019 (COVID-19) pandemic has created large-scale morbidity and mortality, burdened healthcare infrastructures, and created inequitable availability, access, distribution, and uptake of COVID-19 vaccines. As of July 29, 2022, the World Health Organization (WHO) has reported a total of 574 million COVID-19 cases globally and 6.4 million deaths [1]. In Canada, there have been over four million cases and over 42,000 deaths as of July 29, 2022 [2]. Ontario has reported nearly 1.4 million cases and over 13,000 COVID-19-related deaths [2]. Despite the severity of COVID-19, misinformation, cultural beliefs and practices, and a lack of trust in the healthcare system are the largest contributors to vaccine hesitancy. According to the WHO, the main reasons why some individuals decide not to get vaccinated are multifaceted and range from complacency, lack of trust, and barriers and inconveniences in acquiring vaccines [3].

Research focusing on the role of ethnicity, and immigration status in COVID-19 outcomes shows that certain ethnicities, minority groups, and immigration status are at an increased risk of acquiring COVID-19 and experiencing worse clinical outcomes compared to White individuals [4-6]. Additionally, they are more likely to be working under more vulnerable conditions, like in public-facing, service-based occupations and/or self-employed, which provides them with less ability for social distancing and higher rates of infections and hospitalizations [5,6]. Patel et al. showed that, in the United Kingdom (UK), the death rates among Bangladeshi individuals infected with COVID-19 were twice as high as White British individuals [7]. Also, the all-cause mortality (death from any cause) was three times higher in Asian men than expected for this period of 2019-2020 based on death rates in 2014-2018, whereas it was 1.7 times higher in White men [7,8]. The significant prevalence of existing comorbidities in Black, Indigenous, and People of Colour (BIPOC) individuals such as diabetes, hypertension, and cardiovascular diseases [9,10] and other factors such as occupation and household composition [11] increases the risk of severe disease and mortality due to COVID-19 compared to Caucasian individuals.

Despite scientific research pointing to COVID-19 vaccines as being highly effective, hesitancy toward COVID-19 vaccines is significant [12]. A study conducted among United States (US) and UK participants revealed that racial and ethnic minorities were more hesitant toward the COVID-19 vaccine compared to Caucasian individuals [13]. Vaccine hesitancy is not only dangerous for hesitant individuals but also detrimental to society overall. Vaccine hesitancy reduces “herd immunity”, which is “the resistance to the spread of an infectious disease within a population that is based on pre-existing immunity” [14]. In a country like Canada with a minority population exceeding 7 million (in 2016), vaccine hesitancy in BIPOC individuals poses a significant issue that needs to be addressed immediately [15]. The purpose of this study was to identify key barriers and concerns that contribute to vaccine hesitancy in the BIPOC community. Findings from this study may be used to introduce and establish policies and programs that target vaccine hesitancy in the BIPOC community.

## Materials and methods

Study design and settings

The study used an online cross-sectional survey approach to recruit BIPOC and Caucasian participants through social media networks between August 13, 2021, and September 30, 2021. The survey was distributed electronically through community and religious organizations and social media platforms within the Greater Toronto Area (GTA) region in Ontario, Canada. The Institutional Review Board of Toronto Metropolitan University approved the study (approval number: REB 2021-298). The survey consisted of five sections that covered questions linked to participants' sociodemographic characteristics, vaccine knowledge, vaccine status, vaccine intention, and preference.

Participants

A total of 1491 participants (≥ 18 years) completed the survey (see Appendix A). Due to COVID-19-imposed lockdowns and restrictions, a community-based national sampling survey was not feasible. Therefore, an online survey was created and used to collect data for the study. Participants were categorized as BIPOC and Caucasian groups to allow for comparison based on their ethnicities. BIPOC categories were designated according to Statistics Canada and included African American, Arab, Indigenous, East Asian, South Asian, and West Asian participants while Caucasian categories included European, White Canadian, Italian, Latin American, Mixed, and South American [16]. A total of 551 BIPOC and 927 Caucasian respondents were recruited. The research team assumed that 50% of desired responses of knowledge, attitude, and practice (KAP), with a 5% level of significance, and a 3.0% margin of error would yield a total sample size of 1068 to achieve 80% power. Thirteen participants refused to share their ethnicities on the survey and were excluded from the analyses as a result. The study sample was drawn from different cities across Ontario, Canada.

Data collection procedure

A standardized, anonymous, structured online questionnaire/survey tool was developed and adapted from a Malaysian research study (a validated questionnaire that was used to assess vaccine hesitancy among adults) to gather data from respondents [17]. The online questionnaire was piloted before the actual data collection by taking a sample of 30 participants who assessed the content of the questions and their acceptability and comprehensibility, and the results were not included n in the analysis. Participants were provided with a written consent form prior to completion of the online questionnaire. The consent form included a short overview of the context, purpose, procedures, risks, and benefits of participation. Participants were informed that all data would remain anonymous and were told how the data would be used and stored, that participation is voluntary and confidential, and that any participants could remove themselves from the study at any point before they clicked the submit button. The survey on SurveyMonkey (SurveyMonkey Inc., San Mateo, California, United States) was distributed electronically through community partner agencies, religious organizations, and social media platforms across Ontario, Canada, and as hard copies. The completion time for the 20-question survey was approximately five to eight minutes. An incentive for a chance to win $100 was attached to the survey to encourage participation.

Data collection tools

The survey questionnaire was adapted from Syed Alwi et al.'s COVID-19 vaccine acceptance research study and consisted of several multiple-choice questions that inquired about participant’s sociodemographic characteristics, vaccine status, intention and preferences, and general and specific concerns regarding COVID-19 vaccines [17]. The questionnaire also asked about the participant’s knowledge about the COVID-19 vaccine and clinics, and the responses to those questions were in dichotomous ‘yes’ or ‘no’ format (multiple choices). In addition, the attitudes and behavior questions were Likert-scale. To understand potential underlying vaccine concerns leading to hesitancy, participants were asked what general concerns they had regarding regular vaccines and what specific concerns they had about COVID-19 vaccines. Also, the sources of vaccine information and the participants’ behavior toward the COVID-19 vaccine were explored (Appendix A). A reliability test was conducted for questions 6 and 7 and questions 10 to 12 to determine the internal consistency of items listed measuring COVID-19 vaccine concerns and attitude, respectively, resulting in Cronbach’s alpha of more than 0.8 [18].

Data analysis

Descriptive analyses were conducted to establish relationships between sociodemographic characteristics, vaccine status, intention, and preferences, general and specific concerns, sources of information, and attitudes and behaviors towards COVID-19 vaccines. Descriptive statistics were presented as percentages (%) for categorical variables. A chi-square analysis was performed for the respondents' sociodemographic characteristics and the COVID-19 vaccine decisions. Further analyses were conducted to identify predictors of COVID-19 vaccine concern, and attitude using multiple logistic regression for BIPOC and Caucasian respondents. 

All analyses were completed using IBM SPSS Statistics for Windows, Version 23.0 (Released 2015; IBM Corp., Armonk, New York, United States), which was chosen for its robust capabilities in handling categorical data analysis. The level of significance was set at 5% for all analyses. Graphical representation of the data was performed using Microsoft Excel (Microsoft Corporation, Redmond, Washington, United States).

## Results

A total of 1491 participants completed the survey out of which 551 identified as BIPOC, 927 were Caucasian and 13 participants refused to disclose their race (and were not included in data analyses). Therefore, data analysis was performed on 1478 participants.

Demographic characteristics

Respondents were 18 years and older and the average age of respondents was 30.1 years old. Among the BIPOC category, the average age was 31.9, and in the Caucasian categories, it was 29.1 years. Among BIPOC participants, women respondents were slightly higher (49.9%) than men (46.5%), and among Caucasian participants, most were men (75.7%). Most of the respondents were Caucasian (62.7%). BIPOC (37.8%) respondents included individuals who identified themselves as African American (24.5%), Arab (5.8%), East Asian (19.1%), Indigenous (14.0%), South Asian (33.4%), and West Asian (3.3%). The sociodemographic characteristics of the participants are shown in Table 1.

**Table 1:** Sociodemographic characteristics of the participants by age, gender, and race BIPOC: Black, Indigenous, and People of Colour; COVID-19: coronavirus disease 2019

Variables	BIPOC participants (N=551), n(%)	Caucasian participants (N=927), n (%)	Total (N=1478), n (%)
Age (years)			
18-25	95 (17.2)	141 (15.2)	236 (16.0)
26-35	208 (37.7)	520 (56.1)	728 (49.3)
36-45	135 (24.5)	177 (19.1)	312 (21.1)
46-55	61 (11.1)	42 (4.5)	103 (7.0)
56 and over	47 (8.5)	47 (5.1)	94 (6.4)
Prefer not to answer	5 (0.9)	0 (0)	5 (0.3)
Mean age in years	31.9	29.1	30.1
Gender			
Man	256 (46.5)	702 (75.7)	958 (64.8)
Woman	275 (49.9)	209 (22.5)	484 (32.7)
Other	16 (2.9)	4 (0.4)	20 (1.4)
Prefer not to answer	4 (0.7)	12 (1.3)	16 (1.1)
BIPOC subgroups			
African American	135 (24.5)		
Arab	32 (5.8)		
East Asian	105 (19.1)		
Indigenous	77 (14.0)		
South Asian	184 (33.4)		
West Asian (e.g., Iranian, Afghan)	18 (3.3)		
Prefer not to answer	0 (0)		
COVID-19 vaccine status			
First dose only	118 (21.4)	367 (39.6)	485 (32.8)
Both doses	279 (50.6)	337 (36.4)	616 (41.7)
No, but plan on taking it	33 (6.0)	160 (17.3)	193(13.1)
No, and do not plan on taking it	94 (17.1)	39 (4.2)	133 (9.0)
Unsure	23 (4.2)	22 (2.4)	45 (3.0)
Prefer not to answer	4 (0.7)	2 (0.2)	6 (0.4)
COVID-19 vaccine intention			
Yes	318 (57.7)	776 (83.7)	1094 (74.0)
No	106 (19.2)	37 (4.0)	143 (9.7)
Maybe	31 (5.6)	61 (6.6)	92 (6.2)
Prefer not to answer	96 (17.4)	53 (5.7)	149 (10.1)
COVID-19 vaccine preference			
AstraZeneca/COVISHIELD	83 (15.1)	272 (29.3)	355 (24.0)
Janssen (Johnson & Johnson) Moderna	91 (16.5)	199 (21.5)	290 (19.6)
Pfizer-BioNTech	188 (34.1)	214 (23.1)	402 (27.2)
No preference/not sure	14 (2.7)	13 (1.4)	28 (1.9)
Other (Sinopharm/Novavax)	2 (0.4)	0	2 (0.1)
Prefer not to answer	173 (31.4%)	229 (24.7)	402 (27.2)

Vaccine status, intention, and preference

A higher proportion of BIPOC respondents received both doses (50.6%) compared to the Caucasian respondents (36.4%) (p <0.001). However, out of unvaccinated respondents, a statistically significant (p<0.001) higher percentage of BIPOC respondents did not plan on taking the vaccine (17.1% vs 4.2%) compared to Caucasian respondents. Vaccine intention was also lower among BIPOC respondents (57.7% vs 83.7% and p<0.001). BIPOC respondents most preferred Pfizer-BioNTech (34.1%) whereas Caucasian respondents preferred AstraZeneca (29.3%). Both groups had a low percentage of respondents who had no vaccine preference (2.7% BIPOC vs 1.4% Caucasian; Table 1).

Concerns regarding vaccines in general and COVID-19 vaccines in particular

**Figure 1:**
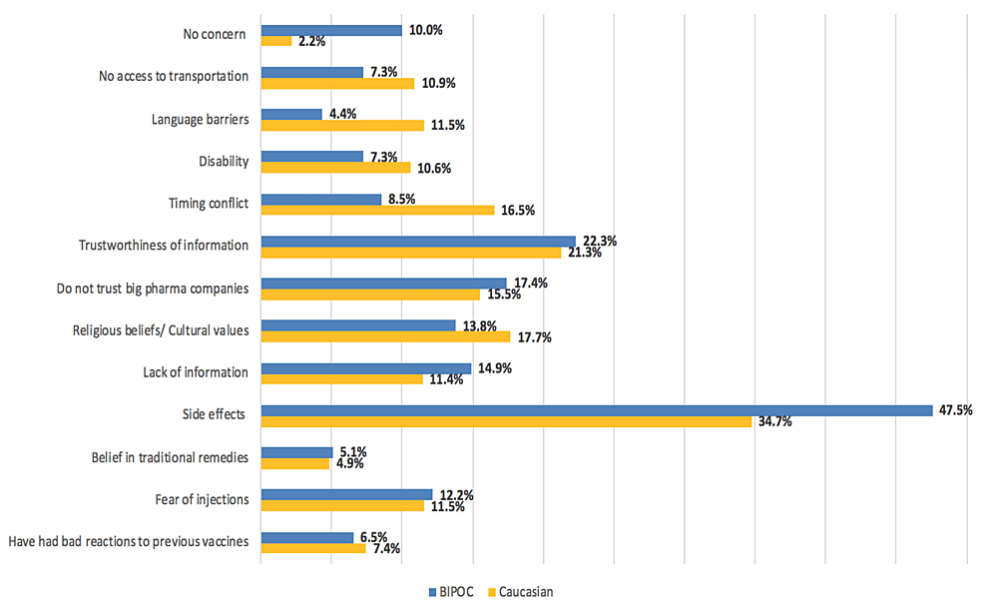
General concerns regarding vaccination among BIPOC (N=551) and Caucasian (N=927) respondents “Question 6: What are your general concerns regarding vaccinations?" was a multiple-response question where participants had the option to select more than one response. BIPOC: Black, Indigenous, and People of Colour

The biggest concerns BIPOC and Caucasian respondents had with general vaccinations were side effects (47.5% vs 34.7%, respectively) and trustworthiness of the information (22.3% vs 21.3%, respectively). Between BIPOC and Caucasian respondents, mistrust of big pharmaceutical companies (17.4% vs 15.5%, respectively), a lack of information (14.9% vs 11.4%, respectively), and religious and cultural beliefs (13.8% vs 17.7%, respectively) were also identified as general concerns, but less frequently. Caucasian respondents included timing conflicts as another major concern (8.5% BIPOC vs 16.5% Caucasian; Figure 1).

**Figure 2:**
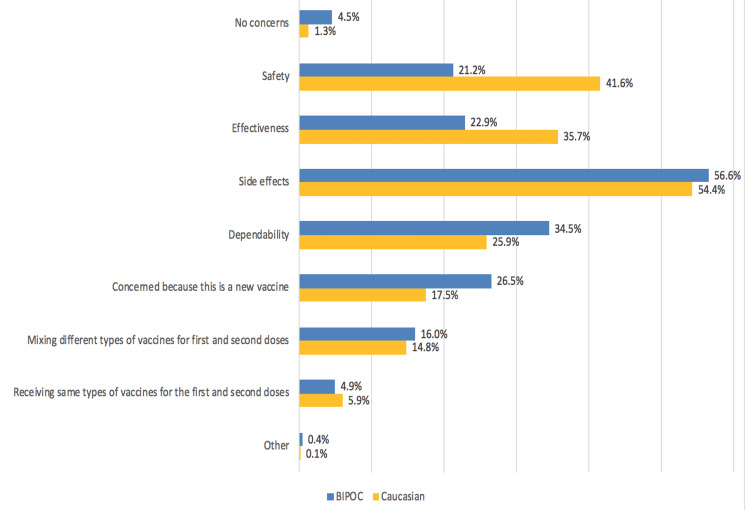
Specific concerns regarding COVID-19 vaccination among BIPOC (N=551) and Caucasian (N=927) respondents “Question 7: What are your specific concerns regarding the COVID-19 vaccine?" was a multiple-response question where participants had the option to select more than one response. BIPOC: Black, Indigenous, and People of Colour

The biggest concern BIPOC and Caucasian respondents had with COVID-19 vaccines specifically were also side effects (56.6% vs 54.4%, respectively). BIPOC respondents identified dependability (34.5% vs 25.9%) and COVID-19 being a new vaccine (26.5% BIPOC vs 17.5% Caucasian) as the next biggest concerns following side effects. However, Caucasian respondents identified safety (21.2% BIPOC vs 41.6% Caucasian) and effectiveness (22.9% BIPOC vs 35.7% Caucasians) as their biggest concerns following side effects (Figure 2).

Most participants had at least one concern related to the COVID-19 vaccine. Overall, there were very few participants from both groups who did not have any concerns (10.0% BIPOC vs 2.2% Caucasians).

KAP regarding COVID-19 vaccination

The primary source of information regarding COVID-19 vaccines and clinics for both BIPOC and Caucasian participants was the media (116.3% vs 95.8%, respectively) (Table 2). Few BIPOC respondents were keen (27.6 %) and positive (36.8%) towards the COVID-19 vaccine in comparison to Caucasian respondents, who were keener (40.1%) and slightly more positive (38.8%). A higher percentage of BIPOC respondents were against vaccinations compared to Caucasians (16% vs 1.2%) (Table 3).

**Table 2:** Knowledge about COVID-19 vaccines and clinics BIPOC: Black, Indigenous, and People of Colour

Variables		BIPOC (N=551), n (%)	Caucasian (N=927), n (%)	Total (N=1478), n (%)
Source of vaccine information (Multiple responses)	Physicians/Family Doctor	147 (26.7)	423 (45.6)	570 (38.6)
	Religious Leaders/ Community Leaders/ Politicians	151 (27.4)	300 (32.4)	451 (30.5)
	Media (print, electronic, news)	551 (100.0)	888 (95.8)	1439 (97.4)
	Friends, family, neighbor and work	239 (43.4)	232 (25.0)	471 (31.9)
	None of the above	16 (2.9)	24 (2.6)	40 (2.7)
	Prefer not to answer	5 (0.9)	9 (1.0)	14 (0.9)
	Other	0 (0)	0 (0)	0 (0)
Source of vaccine clinic information (Multiple responses)	Physicians/Family Doctor	149 (27.0)	319 (34.4)	468 (31.7)
	Religious Leaders/Community Leaders/ Politicians	166 (30.1)	236 (25.5)	402 (27.2)
	Media (print, electronic, news)	551 (100.0)	891 (96.1)	1442(97.6)
	Friends, family, neighbor and work	234 (42.5)	217 (23.4)	451 (30.5)
	None of the above	23 (4.2)	22 (2.4)	45 (3.0)
	Prefer not to answer	3 (0.5)	7 (0.8)	10 (0.7)
	Other	4 (0.7)	1 (0.1)	5 (0.3)

**Table 3:** Attitude and behavior towards COVID-19 vaccine BIPOC: Black, Indigenous, and People of Colour

Variables		BIPOC (N=551), n (%)	Caucasian (927), n (%)	Total (1478), n (%)
Participant’s attitude	Very keen	152 (27.6)	372 (40.1)	524 (35.5)
	Positive	203 (36.8)	360 (38.8)	563 (38.1)
	Neutral	63 (11.4)	137 (14.8)	200 (13.5)
	Uneasy	24 (4.4)	33 (3.6)	57 (3.9)
	Against it	88 (16.0)	11( 1.2)	99 (6.7)
	Don’t know	14 (2.5)	7 (0.8)	21 (1.4)
	Prefer not to answer	7 (1.3)	7 (0.8)	14 (0.90)
Advice to family and friends	Strongly encourage them	236 (42.8)	442 (47.7)	678 (45.9)
	Encourage them	122 (22.1)	287 (31.0)	409 (27.7)
	Not say anything to them	80 (14.5)	103 (11.1)	183 (12.4)
	Ask them to delay getting the vaccination	17 (3.1)	63 (6.8)	80 (5.4)
	Suggest that they do not get the vaccination	79 (14.3)	16 (1.7)	95 (6.4)
	Don’t know	13 (2.4)	7 (0.8)	20 (1.4)
	Prefer not to answer	4 (0.7)	9 (1.0)	13 (0.9)
Importance of vaccination	Very important	252 (45.7)	480 (51.8)	732 (49.5)
	Fairly important	87 (15.8)	221 (23.8)	308 (20.8)
	Important	63 (11.4)	97 (10.5)	160 (10.8)
	Slightly important	16 (2.9)	74 (8.0)	90 (6.1)
	Not at all important	88 (16.0)	19 (2.0)	107 (7.2)
	No opinion	38 (6.9)	18 (1.9)	56 (3.8)
	Prefer not to answer	7 (1.3)	18 (1.9)	25 (1.7)

Factors associated with COVID-19 vaccine concern and attitude

The results of the multiple logistic regression model showed that two independent predictors were significantly associated with BIPOC and Caucasian respondents' COVID-19 vaccine preference attitude. In the model, the dependent variable was respondents (Caucasian as 0 and BIPOC as 1) and the independent variables were age, gender, vaccine suggestions to friends and family, vaccine attitude towards vaccination, type of vaccine preferred, and intent to receive the COVID-19 vaccine (Model 1). The model shows a good fit from Omnibus Tests of Model Coefficients (p =0.001).

Nagelkerke R2 is a coefficient of determination that measures how well a statistical model predicts an outcome. R2 is a statistical measure of the proportion of variation in the dependent variable that the model predicts. Overall, the model explained 22% (Nagelkerke R2) of the variance in BIPOC and Caucasian’s COVID-19 vaccine preference attitude. Only age, gender, vaccine suggestions to family and friends, and type of vaccine preference were statistically significant. Caucasian and BIPOC respondents had different preferred vaccines. Also, more male Caucasian participants responded on the vaccine than male BIPOC participants (Table 4).

**Table 4:** Multiple logistic regression model 1 COVID-19: coronavirus disease 2019

		B	Exp(B)	95% CI for EXP(B)		
				Lower	Upper	Sig.
	Age					
	18-25 years		1			< .001
	26-35 years	-.325	.722	.062	8.457	.796
	36-45 years	-1.603	.201	.018	2.308	.198
	46-55 years	-1.354	.258	.022	3.019	.281
	56-65 years	-.304	.738	.059	9.183	.813
	66-75 years	-1.047	.351	.027	4.530	.422
	>76 years	-.318	.727	.027	19.627	.850
	Gender					
	Male		1			< .001
	Female	-1.109	.330	.233	.467	< .001
	If my family or friends were thinking of getting the COVID-19 vaccine					
	Suggest that they do not get the vaccination		1			.032
	Ask them to delay getting the vaccination	1.494	4.453	.579	34.264	.151
	Not say anything to them	-.221	.801	.267	2.409	.693
	Encourage them	.182	1.200	.612	2.353	.596
	Strongly encourage them	-.497	.608	.383	.966	.035
	If you needed a vaccine, which do you prefer					
	AstraZeneca/COVISHIELD		1			< .001
	Janssen (Johnson & Johnson)	-1.874	.153	.054	.432	< .001
	Pfizer-BioNTech	-1.256	.285	.099	.822	.020
	Other	-1.072	.342	.121	.966	.043

Another model was run using the dependent variable “want vaccine (1)” or “do not want vaccine (0)", and independent variables were age, gender, taking the COVID-19 vaccine, vaccine suggestions to family or friends, Attitude towards vaccination, type of vaccine preferred, and respondent race (BIPOC and Caucasian). The model also shows a good fit from Omnibus Tests of Model Coefficients (p =0.001). Nagelkerke R2 48.5% of the variation whether respondents “want vaccine” or “do not want vaccine is explained by all the variables included as independent variables in the model. No individual/ independent variables were statistically significant predictors.

## Discussion

This study investigated the challenges and concerns that contribute to vaccine hesitancy in BIPOC individuals in Ontario, Canada. Both BIPOC and Caucasian individuals participated in it. Side effects and dependability were identified as the largest concerns respondents had towards getting the COVID-19 vaccine. Concerns that contributed to vaccine hesitancy in general were side effects, mistrust, and a lack of information about vaccines.

Overall, the survey had a higher representation of Caucasian participants compared to BIPOC participants at 62.7% and 37.3%, respectively. Most of the participants from both groups were 26-35 years of age. In the BIPOC group, females (49.9%) were slightly more than males (46.5%) while majority of the Caucasian respondents were males (75.5%). The study findings were in line with a similar study examining and comparing the attitudes and hesitancy related to influenza vaccine uptake between an equal representation of African American and White American individuals [19]. A higher proportion of BIPOC respondents received both doses (50.6%) compared to Caucasian respondents (36.4%) (p <0.001). Among the vaccinated groups, the Caucasian group was more likely to have "First dose only" or "no plans on taking it" which may suggest that the pace of COVID-19 vaccination was slower than that of BIPOC groups. In addition, BIPOC respondents mostly preferred Pfizer-BioNTech (34.1%) whereas Caucasians preferred AstraZeneca (29.3%). Similarly, Dula and colleagues showed that vaccine acceptance varied with the manufacturer (brand), country of origin, and other aspects [20].

In a few other studies [21-23] in the UK and US, the biggest concern BIPOC and Caucasian participants, both men and women, had with general vaccinations and COVID-19 vaccines were side effects. The reported intention to get the vaccine was notably higher in White participants than in Black participants [23]. On the other hand, in a cross-sectional study across India, the UK, Germany, Italy, and Spain; European respondents cited more concerns regarding the COVID-19 vaccine side effects [24]. 

Trustworthiness and a lack of trust in big pharma companies were identified as the second biggest concerns among the BIPOC participants, regardless of their vaccination status. Compared to White British or Irish respondents, a study in the UK found that more Black or Black British respondents reported their mistrust of vaccines [21]. The hesitancy of BIPOC individuals towards the COVID-19 vaccine has been linked to historical structural racism, unethical research studies, and institutional untrustworthiness [19,25,26]. In contrast, experiences of discrimination in the healthcare system negatively correlated with uptake, perceived side effects, and trust in the vaccine [19]. Another study also highlighted false information, rumors, and conspiracy theories as key causes that undermine trust in COVID-19 vaccinations with social media as its source [27].

BIPOC and Caucasian respondents demonstrated positive attitudes towards vaccinations and identified media as their primary source of information. BIPOC participants identified friends, family, neighbors, and work followed by religious and community leaders and politicians as their secondary sources of information. Similar positive attitudes were shown in several studies in Malaysia [17], Bangladesh [28], China [29], Mozambique [20], and Italy [30] among general people, undergraduate students, Chinese adults, healthcare workers, and the university population, respectively. The secondary sources of information for Caucasian participants were physicians, followed by religious and community leaders and politicians. Di Giuseppe et al. also identified media as the main source of information (63.1%) followed by the Internet (58.1%) and scientific journals (31.1%) [30].

Several individuals who are vaccine-hesitant are heterogeneous; they might agree to some vaccines but remain opposed to others. Studies have also shown that when risk perceptions increase, vaccine acceptance decreases [31] for various reasons such as political, historical, and socio-cultural context, high trust in religious leaders, the role of public health and vaccine policies, contribution of health professionals, knowledge/information about vaccinations, and trust in the healthcare system [24,32]. The proposed methods to address vaccine hesitancy at the population level include incorporating transparency in vaccine policy-making decisions, offering education and information on the procedures that lead to the approval of new vaccines, paying attention to public concerns, and taking public perspectives into account when formulating vaccine policies and programs [33]. It is also necessary that all vaccine-related communication is released in a way that any non-expert audience can understand the information [34].

Increasing vaccine confidence and uptake plays a pivotal role in managing and reducing the effects of COVID-19 infections. While the initial intentions of the vaccine were to assist with controlling the pandemic and accelerating herd immunity, recent studies have indicated herd immunity is not achievable due to variants [35]. Following a few months of vaccination, there is a rapid loss of antibodies and prior infection, or vaccination does not significantly protect against the variant by neutralizing it [35]. However, booster doses of the vaccine have demonstrated short-term effectiveness against variant infections such as Omicron [35]. A study published by the CDC highlighted that the Pfizer and Moderna COVID-19 vaccines' protective effects start to fade four months following a booster dosage [36]. To prevent the spread of Omicron and other subvariants with greater infectious capacity, high levels of vaccination coverage and effectiveness are required [37]. This is necessary to reduce the harmful effects of COVID-19 infections and to create periods of momentary infection protection for a few months for those recently vaccinated. Together these two benefits assist with minimizing the effects of COVID-19 on individuals, protecting the healthcare system and supporting continued efforts to promote economic recovery.

The results of this study indicate the importance of several factors to consider when engaging community members to improve the uptake of COVID-19 vaccines. Increasing the visibility of BIPOC healthcare providers to help engage the population, investing in media literacy, education, and campaigns, using media channels to launch campaigns with scientific research-based information, use of a participatory approach to establish forums where BIPOC individuals are encouraged to address their concerns without fear of judgments and consequences, increase vaccine knowledge and accessibility to BIPOC individuals.

This study had a few limitations including the recruitment of BIPOC participants (recruitment bias). The increased spread of COVID-19, especially the Delta variant at the time of the study, significantly hindered the establishment of relationships and partnerships between the researchers and community agencies. This primarily affected the recruitment of BIPOC participants (including older adults). Robust community engagement efforts through equitable inclusion of religious and community leaders from BIPOC communities are more effective in recruiting BIPOC participants [38]. The White respondents were heavily skewed towards males. Meanwhile, the BIPOC group was almost equally divided into male and female. Therefore, there might be differences in outcomes due to gender (gender bias). Although we had limited in-person interactions, recruitment flyers were posted in the public spaces of the GTA which are frequented by BIPOC communities (see Appendix B). These flyers coupled with the $100 survey incentive helped counter the effects of not conducting in-person recruitment.

The digital delivery of the survey could have posed an accessibility issue for those with less access to technology [32]. The survey’s availability in only English was another limitation, as it prevented some respondents from participating due to the language barrier. Though there were volunteers assisting with approaching Spanish-speaking participants and translating the questionnaire, due to their limited availability, the study experienced challenges assessing several special populations of participants, especially the older BIPOC adults, and may have some generalizability concerns. Thus, it is recommended that there should be future studies to confirm the findings, particularly focused on resources to improve recruitment issues and language barriers.

It is important to recognize the study's strengths as well, which include the recruitment efforts to target and engage the BIPOC community given that there remains very limited understanding of the role of ethnicity in vaccine hesitancy, particularly within Canada. The equal gender composition of males and females in the BIPOC participants assisted with assessing the impact of gender and the specific findings associated with preferences for primary sources of information in the BIPOC community. Together, these findings can assist public health efforts and future vaccine campaigns aimed at increasing vaccination rates within the BIPOC community. Furthermore, the focus may affect how the study’s findings are interpreted. By focusing on the 17.1% who chose "No, and do not plan on taking it" among the BIPOC group, it can be interpreted that a higher percentage of BIPOC respondents did not plan on getting vaccinated (17.1%) compared to Caucasian respondents (4.2%). However, focusing on the BIPOC group who chose "Both doses" (50.6%), it can be said that the BIPOC group is polarized between those who are "actively vaccinated" and those who are "not vaccinated." By dividing the BIPOC group into two subgroups of "Both doses" and "No, and do not plan on taking it" and comparing factors such as "the associated side effects and lack and mistrust of information," it may be possible to find clues to increasing the vaccination rate in the future studies.

On the other hand, the Caucasian group played the role of "reference", but it is noteworthy that the percentage of "First dose only" and "No, but plan on taking it" were higher than those of BIPOC group. Considering "No, but plan on taking it" does not truly mean taking the vaccine, the percentage of those not vaccinated ("No, but plan on taking it" and "No, and do not plan on taking it") for BIPOC groups is 23.1%, and for the Caucasian group, it is 21.5%. Therefore, those not vaccinated are close between both groups. The findings of this study suggest that the pace of vaccination in the Caucasian group is slow and that there is room to look for ways to improve the pace of vaccination in future studies. While this study was focused on improvements for BIPOC groups, the Caucasian group also had room for improvement (the pace of vaccinations). Future studies can be performed to find the improvements and issues regarding the Caucasian group.

Further research can be performed to investigate the reasons why BIPOC individuals prefer family and friends over physicians and community leaders as their primary sources of information. The study can focus on ways to increase awareness of gathering information using more reliable sources. Further research can also study the reason behind why BIPOCs prefer the Pfizer vaccine over the Jansen (Johnson & Johnson) and Moderna vaccines. Similar studies can also be performed to assess vaccine hesitancy in specific BIPOC communities with a larger sample size. This will help understand the racial and ethnic differences in vaccination status, attitudes, and behaviors within targeted BIPOC communities.

## Conclusions

This study provided valuable insight into COVID-19 vaccines in the BIPOC community. The study demonstrated that although BIPOC individuals had a generally positive attitude and believed in the importance of the COVID-19 vaccine, there were increased concerns about the associated side effects and lack and mistrust of information. Effective methods to respectfully address the identified concerns were determined to be using relevant channels such as social media, news, and radio. Findings from this study can be used to design and implement engagement campaigns that can promote vaccine confidence in BIPOC communities not only in Canada but also around the world. 

## Figures and Tables

**Figure 3:**
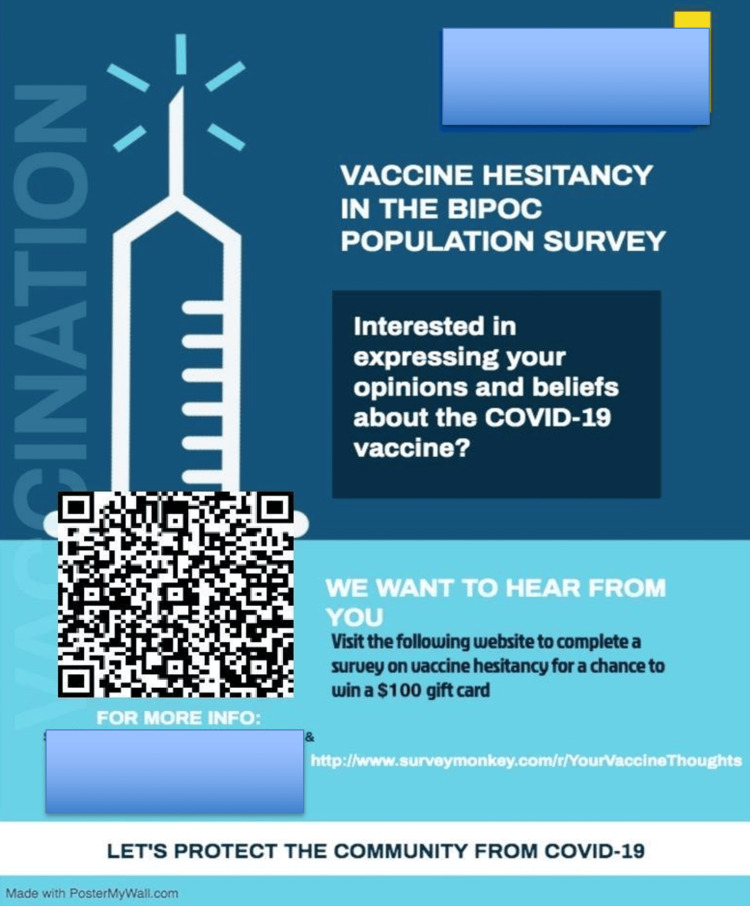
Recruitment flyer 1

**Figure 4:**
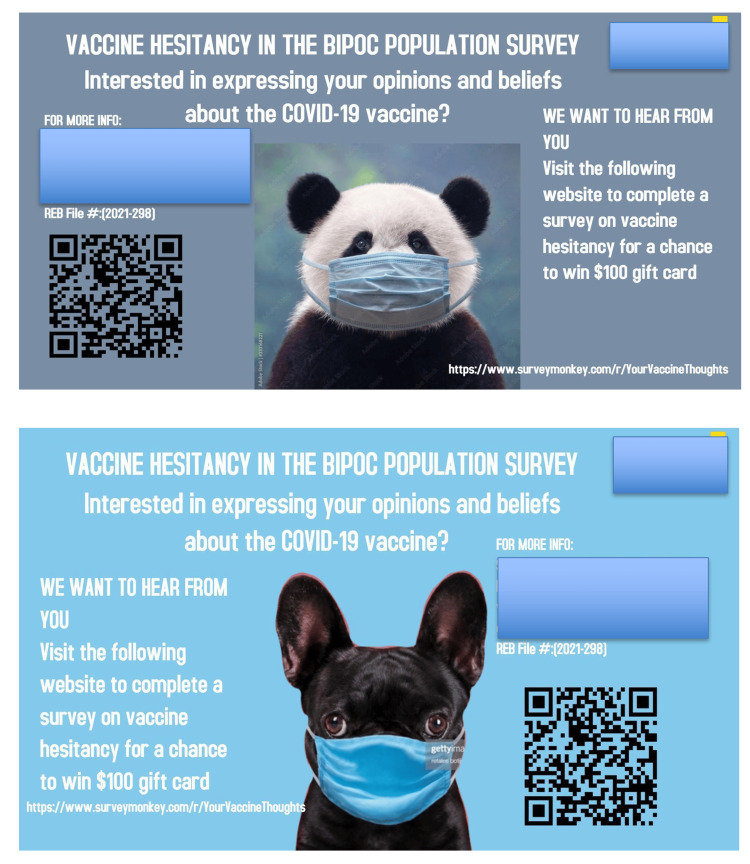
Recruitment flyer 2

## Appendices

Appendix A

**Table 5:** Survey on SurveyMonkey that was distributed to recruit participants

Survey
Dear ­­­­­­participant, My name is …….. I am a faculty member at University in the School of……. I would like to invite you to take part in our research study, which explores Vaccine Hesitancy in BIPOC individuals over 18 years and older.
WHAT YOU ARE BEING ASKED TO DO You are being asked to voluntarily complete this online survey. It involves questions about your opinions and thoughts about the COVID-19 vaccine and the barriers leading to vaccine hesitancy. This survey is to be completed electronically with internet access and should take about approximately 5 to 10 minutes to complete. In order for all of your answers to be collected you must go to the end of the survey and click ‘submit’. This will demonstrate your full consent to participation. We hope to recruit appropriately 2500 participants for this survey. POTENTIAL BENEFITS It is hoped that the research will inform a larger quantitative study in the future directed toward a greater understanding of the barriers leading up to vaccine hesitancy. Information gathered from this survey may contribute towards developing and implementing targeted campaign(s). The Research team cannot guarantee any direct benefits to you. WHAT ARE THE POTENTIAL RISKS TO YOU Some of the survey questions may make you uncomfortable or upset or you may simply wish not to answer some questions. You are free to decline to answer any questions you do not wish to answer or stop participating at any time by closing your browser. If you close your browser before getting to the end of the survey and do not confirm your consent to participate at the end of the survey by clicking the ‘submit’ button your information collected up to that point will not be used. YOUR IDENTITY WILL BE ANONYMOUS The survey is anonymous and as such will not be collecting information that will easily identify you, like your name or other unique identifiers. However, if you choose to provide your email address to participate in the draw, please note that your identity may no longer be anonymous. However, this information will be kept confidential. Although your Internet Protocol (IP) address can be tracked through the survey platform, the researcher/s will not be collecting this information. Your IP address may be observed only to ensure that one individual is not completing the survey multiple times. HOW YOUR INFORMATION WILL BE PROTECTED AND STORED This survey uses SurveyMonkey which is a United States of American (USA) company. Consequently, USA authorities under the provisions of the USA Freedom Act may access the survey data. To further protect your information, data stored by the researcher will be password-protected and/or encrypted. Only the researchers named in this study will have access to the data as collected. Any future publications will include collective information (i.e., aggregate data). Your individual responses (i.e. raw data) will not be shared with anyone outside of the research team. When the research is completed, the researchers will keep the data for up to seven years after the study is over. INCENTIVE FOR PARTICIPATION Participation in this survey is highly valued. Please provide your email address if you are interested in entering a draw for a chance to win a $100 gift card. Please note that by providing your email address for the draw, your identity may no longer be anonymous. However, this information will be kept confidential. If you would like to complete the survey but not participate in the draw, please skip the email address question. DATA DISSEMINATION Once the survey is closed, the Research team will analyze the data collected from this survey. The analyzed data may be used to create a targeted campaign(s) that addresses COVID-19 vaccine hesitancy and key concerns. If you wish to receive a copy of the findings of this study, please indicate so in question 18 of this survey. YOUR RIGHTS AS A RESEARCH PARTICIPANT Participation in research is completely voluntary and you can withdraw your consent at any point up to clicking the submit button at the end of the survey. By consenting to participate you are not waiving any of your legal rights as a research participant.
QUESTIONS
If you have any questions about this research, please feel free to contact any of the following researchers on this study. ………………………….. …………………………….. …………………………….. ……………………………… If you have any questions about your rights or treatment as a research participant in this study, please contact the ……………… Ethics Board at ………………….. Please print a copy of this page for your future reference. By checking the box below, I am consenting to participate in this survey. ☐ I consent to participate in this survey.
Have you received the COVID-19 vaccine? Check only one box.
☐Yes, I have taken the FIRST dose of the COVID-19 vaccine
☐Yes, I have taken BOTH doses of the COVID-19 vaccine
☐No, I have not taken the COVID-19 vaccine and plan on taking it
☐No, I have not taken the COVID-19 vaccine and do not plan to take it
☐Unsure
☐Prefer not to answer
If you selected “No” or “Unsure” on previous question, tell us why Check all that apply.
☐I took part in a vaccine trial
☐Religious reasons
☐Personal belief/ philosophical reasons
☐Pregnancy/ breastfeeding
☐Concerned about the long-term side effects
☐Concerned about adverse reaction
☐Do not know enough about it
☐Illness/ Medication
☐Do not think it is available to me
☐Do not think it is necessary
☐Do not think it will work
☐Prefer not to say
☐I am against vaccines in general
☐Other:
Do you intend on receiving the COVID-19 vaccine? Check only one box.
☐Yes
☐No
☐Maybe
Are you aware of the vaccine clinics in your area, if so how did you hear about them?
☐Physicians/Family Doctor
☐Religious Leaders
☐Community Leaders / Politicians
☐ Research on the internet
☐Print media (newspapers, brochures, flyers etc. )
☐Social media platforms (Facebook, Twitter, Instagram, Tik Tok etc.)
☐Friends, Family and neighbour etc.
☐News
☐None of the above
☐Prefer not to answer
☐Other:
Has your Physician spoken to you about the vaccine?
☐Yes
☐No
☐No family Physician
What are your general concerns regarding vaccinations? Check all that apply.
☐No access to transportation
☐Language barriers
☐Disability
☐Timing conflict
☐Trustworthiness of information
☐Do not trust big pharma companies
☐Religious beliefs
☐Cultural values
☐Lack of information
☐Side effects
☐Belief in traditional remedies
☐Fear of injections
☐Have had bad reactions to previous vaccines
☐Other:
What are your specific concerns regarding the COVID-19 vaccine? Check all that apply.
☐Safety
☐Effectiveness
☐Side effects
☐Dependability
☐Concerned because this is a new vaccine
☐Mixing different types of vaccines for first and second doses
☐Receiving same types of vaccines for the first and second doses
☐Other:
Do you have a vaccine preference? Check only one box.
☐Yes
☐No
If “Yes” on previous question, which type of vaccine do you prefer? Check only one box.
☐AstraZeneca/COVISHIELD
☐Janssen (Johnson & Johnson)
☐Moderna
☐Pfizer-BioNTech
☐Other:
I would describe my attitude towards receiving the COVID-19 vaccine as Check only one box.
☐Very keen
☐Positive
☐Neutral
☐Uneasy
☐Against it
☐Don’t know
☐Prefer not to answer
If my family or friends were thinking of getting the COVID-19 vaccine, I would: Check only one box
☐Strongly encourage them
☐Encourage them
☐Not say anything to them
☐Ask them to delay getting the vaccination
☐Suggest that they do not get the vaccination
☐Don’t know
☐Prefer not to answer
Taking the COVID-19 vaccination is: Check only one box.
☐Very Important
☐Fairly Important
☐Important
☐Slightly Important
☐Not at all Important
☐No opinion
☐Prefer not to answer
I get my vaccine information through: Check all that apply.
☐Physicians/Family Doctor
☐Religious Leaders
☐Community Leaders / Politicians
☐ Research on the internet
☐Print media (newspapers, brochures, flyers etc. )
☐Social media platforms (Facebook, Twitter, Instagram, Tik Tok etc.)
☐Friends, Family and neighbour etc.
☐News
☐None of the above
☐Prefer not to answer
☐Other:
What is your age? * Check only one box.
☐18-25
☐26-35
☐36-45
☐46-55
☐56-65
☐66-75
☐76 and over
☐Prefer not to answer
What is your gender? Check only one box.
☐Man
☐Woman
☐Non-binary
☐Transgender
☐Intersex
☐Gender non-conforming
☐Prefer to specify___________
What are the first 3 characters of your postal code?
I identify myself as: Check only one box.
☐Arab
☐Black
☐Chinese
☐Filipino
☐Indigenous
☐Japanese
☐Korean
☐Latin American
☐South Asian (e.g., East Indian, Pakistani, Sri Lankan)
☐Southeast Asian (e.g., Vietnamese, Cambodian, Laotian, Thai)
☐West Asian (e.g., Iranian, Afghan) ☐White
☐Prefer not to answer
☐Other:
Do you wish to participate in the draw for a chance to win a $50.00 gift card? If “yes”, please provide email address in Question 20.
☐Yes
☐No
Do you wish to receive a copy of this survey’s findings to the email address listed below?
☐Yes
☐No
What is your email address (optional) _________________________________________

Appendix B
